# Employing molecular beacons to assess in vitro transcription with single-molecule resolution

**DOI:** 10.1038/s41598-026-55220-6

**Published:** 2026-06-10

**Authors:** Vadim G. Bogatyr, Fatema-Zahra M. Rashid, Andreas S. Biebricher, Wilhelm T. S. Huck, Gijs J. L. Wuite

**Affiliations:** 1 Department of Physics and Astronomy, Physics of Living Systems, VU University Amsterdam, De Boelelaan 1100, 1081HV Amsterdam, The Netherlands; 2 Institute for Molecules and Materials, Radboud University, Heyendaalseweg 135, 6525 AJ Nijmegen, The Netherlands

**Keywords:** Fluorescence microscopy, Transcription, Bottom-up synthetic biology, RNA, Single-molecule, Molecular beacons, Biochemistry, Biological techniques, Biotechnology, Cell biology, Molecular biology

## Abstract

Transcription is a vital cellular process in which RNA polymerase produces messenger RNAs (mRNA) from a DNA template. Many transcription systems have been developed to reproduce this process in vitro and in the confined environment of giant unilamellar vesicles (GUVs). However, these systems and the studies employing them often use DNA concentrations significantly higher than those found in natural cells, which typically contain a single DNA copy. In this work, we introduce single-molecule in vitro transcription (smIVT) that overcomes this limitation, enabling the visualization and tracking of individual mRNA molecules both in solution and within GUVs. We achieved this by employing a molecular beacon—a quenched RNA or DNA hairpin probe that becomes fluorescent after binding to the transcript mRNA—and template DNA encoding 32 repeats of the molecular beacon binding sequence. With these, we ensured a signal-to-noise ratio sufficient to detect single mRNAs. We use smIVT to compare the performance of various commercially-available in vitro transcription kits, and to quantitatively describe single molecule transcription inside GUVs. We establish smIVT as a remarkable technique for scrutinizing in vitro transcription with single-molecule resolution.

**Supplementary Information:**

The online version contains supplementary material available at 10.1038/s41598-026-55220-6.

## Introduction

 Transcription ensures the reliable transfer of information from DNA to newly synthesized messenger RNA (mRNA) which is used for protein expression. Transcription is an essential step in the central dogma of molecular biology and no synthetic cell will function adequately without its successful incorporation. Hence, much effort in the [synthetic] biology field is focused on reconstituting transcription. One of the simplest transcription machineries found in nature is that of the T7 bacteriophage^1^. T7 RNA polymerase (T7 RNAP) is a single macromolecular protein unit^2^ that is characterized by outstanding promoter-specificity^3^, speedy transcription rate^4^, and high fidelity^5^. It is natively compatible with *Escherichia coli*, T7 phages’ natural target and molecular biology’s workhorse. These factors have solidified T7 RNAP as a go-to solution for reconstituted transcription.

With the demand for in vitro generated mRNA steadily increasing, T7 RNAP has found many promising applications, from vaccine production^6^ to gene regulation^7^. Here, we emphasize synthetic cell research, particularly the approach of modifying and coupling existing biological systems to produce a minimal self-sustaining and reproducing unicellular organism. Owing to the extensive use of T7 RNAP for in vitro transcription, an abundance of T7 RNAP-based transcription kits are commercially available promising high RNA yields, no RNase contamination, and optimized reaction buffers. However, the composition of the reagents of the kits are sometimes not disclosed due to trade secrets. Thus, it is non-trivial to narrow down the best commercial T7 RNAP-based transcription kit for use in a synthetic system.

T7 RNAP, along with the translation machinery from *E. coli*, comprise a minimal component, cell-free protein expression system referred to as PURE (*p*rotein synthesis *u*sing *r*ecombinant *e*lements)^8,9^. Commercially-available and home-made PURE mixes have been successfully encapsulated inside giant unilamellar vesicles (GUVs)^10^ to produce cytoskeletal elements^11^, synthesize lipids^12^, and reconstitute cell division^13^. Encapsulation of the PURE system in GUVs has been used to mimic synthetic cells carrying genetic material and transcription-translation machinery. The assembly has been used to track mRNA and protein (YFP) production in real time^14,15^. However, these studies have been done with hundreds or thousands of copies of DNA template encapsulated within the GUV. An approach to studying transcription dynamics within GUVs enclosing one or two copies of DNA templates – as is typical in most living cells – is currently missing. As such, several essential related questions remain unresolved; for example, what are the caveats of using low pM DNA concentration for reconstituted in vitro transcription-translation? What would the effects of the stochastic single-molecule behavior of T7 RNAP be? Can the energy supplies of the reconstituted transcription-translation machinery sustain a reaction over hours and even days? What technical solutions are available to reliably track single mRNA and proteins as they are transcribed and translated?

In this work, we present single molecule in vitro transcription (smIVT) using molecular beacons to visualize single mRNAs. Molecular beacons (MBs) are single-stranded RNA or DNA molecules 20–50 nucleotides long, functionalized by a dye and a quencher at opposite ends. MBs fold into a hairpin by a self-pairing stem region typically 3–5 bp long. In the hairpin conformation, the proximity of the quencher to the dye ensures little to no fluorescence. Unfolding of the hairpin spatially separates the quencher and dye, resulting in a fluorescent signal. Nucleic acid hairpins exist in a thermal equilibrium between the folded and unfolded states. Free ssDNA or RNA in solution carrying a binding site for the MB stabilize its unfolded state, and hence its fluorescent signal^16–19^. The fluorescent signal for tracking individual mRNA molecules can be enhanced by increasing the number of MB-complementary repeats on the transcript. A comparison of smFISH and MB mRNA localization in vivo showed that four MB-complementary repeats allow precise and reliable localization^20^.

Here, we present data to establish molecular beacon-based smIVT as a remarkable technique for investigating in vitro transcription at single-molecule resolution. We use MBs to visualize mRNA carrying 32 target sequences to allow for sufficient signal-to-noise ratio and compare it to the traditional Spinach aptamer-DHFBI technique for visualizing mRNA. We show that smIVT, with mRNA detected using molecular beacons, reproduces expected transcription activity dependencies on DNA concentration and temperature. We demonstrate the utility of the assay by comparing eight commercially-available T7 RNAP-based transcription kits and identifying the best-performing kits. Finally, we showcase how smIVT resolved single mRNAs in GUVs. The assay we describe here is an essential step to understanding transcription in a synthetic cell.

## Results

### Molecular Beacons as prospective single-molecule mRNA probes

To develop a single-molecule transcription assay suitable for synthetic cells, we opted to work with DNA concentrations that correspond to single DNA copies per “synthetic cell”. Assuming an average GUV size of 10 μm, which is the typical size we obtained using cDICE^10^ and the inverted emulsion method for GUV production^21,22^(Supplementary Fig. S1), this corresponds to 1-100 pM DNA—a concentration range 10- to 1000-fold lower than typically used for in vitro transcription (IVT) assays.

**Fig. 1:**
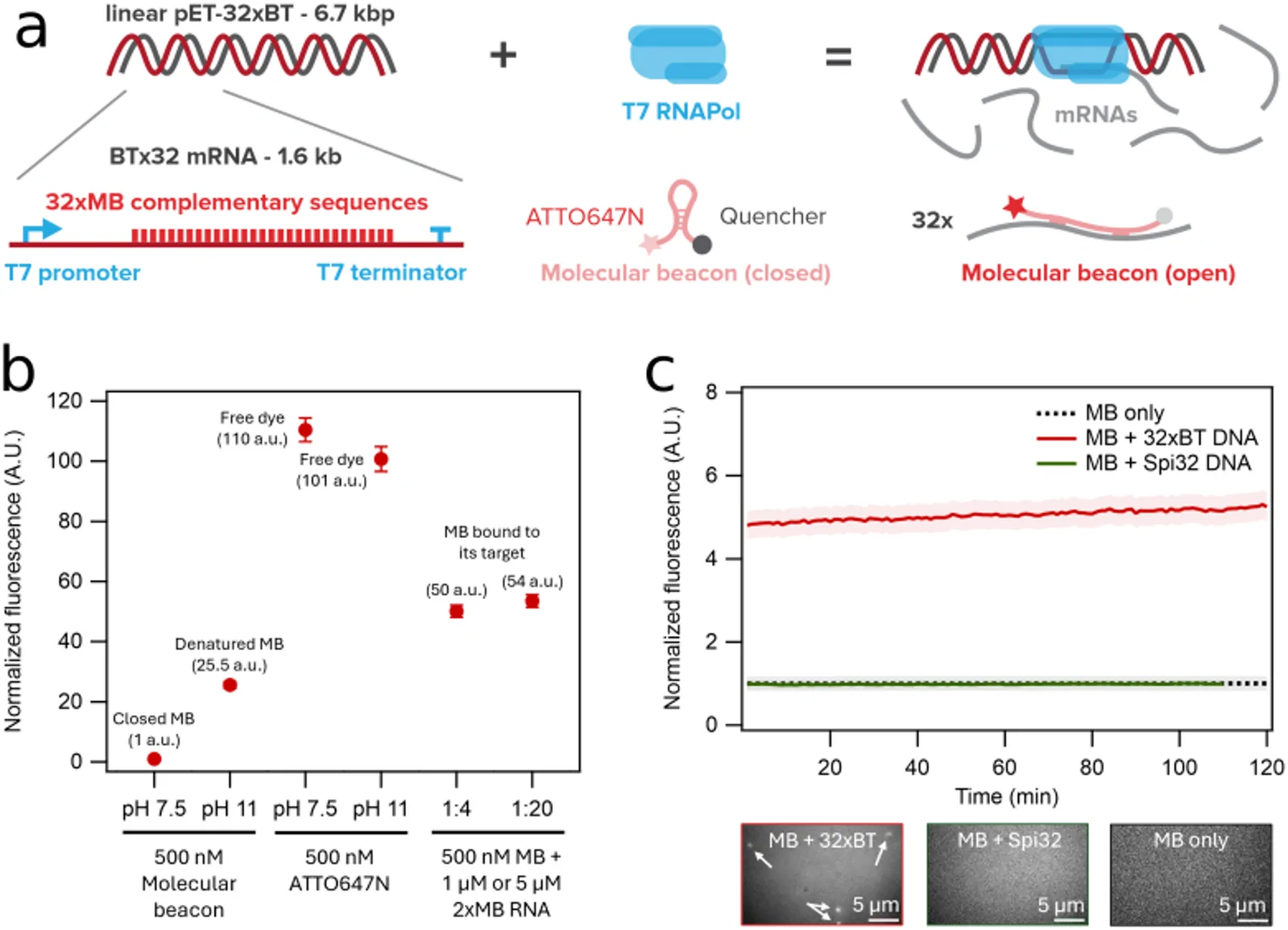
Visualizing mRNA using molecular beacons (MBs). (**a**) Transcription of linearized pET-32xBT with T7 RNAP produces an mRNA carrying 32 MB binding sites. Opening of the molecular beacon hairpin and its hybridization onto its target sites on the mRNA separates the ATTO647N fluorophore from the Iowa Black RQ quencher producing a fluorescence signal. (**b**) In the presence of 4- to 20-fold excess target sites, the bulk fluorescence signal of the MBs used in our study increases up to 54-fold. (**c**) The MBs used in our study are specific for 32xBT mRNA that encodes their target sites. An IVT reaction with 32xBT DNA and MBs had a ~ 5-fold higher normalized fluorescence signal compared to a negative control reaction with only MBs, while the fluorescence signal of the IVT reaction with Spi32 DNA that encodes 32 Spinach aptamers was indistinguishable from the negative control. Diffusive, bright fluorescent spots corresponding to MB-bound transcripts were observed in IVT reactions with 32xBT DNA, but not with Spi32 DNA.

Choosing the proper fluorescent probe is crucial to achieving single mRNA detection. Molecular beacons are promising candidates for this role. They are bright, sequence-specific, and activate upon binding their target. In an earlier work, MBs were used in a bulk experiment with plasmid pET-32xBT that encodes 32 repeats of the MB target sequence to assess macromolecular crowding effects on transcription^23^. Inspired by this, we employ a 2’-O-methylated RNA molecular beacon functionalized with ATTO647N dye on one end and Iowa Black RQ quencher on the other. Methylation of the MB resists nuclease degradation^24^, and improves MB-mRNA stability^19^. The transcription template is provided by linearized pET-32xBT plasmid (32xBT DNA). 32xBT DNA contains a T7 promoter and terminator; thus, T7 RNAP is expected to transcribe an approximately 1.6 kb-long mRNA containing 32 MB target sites with spacers (Fig. 1a). Multiple molecular beacon target sites ensure higher brightness and a better S/N ratio.

To test how efficiently ATTO647N is quenched in the MB, we compared the fluorescence of 500 nM MB (Fig. 1b) to that of 500 nM ATTO647N free dye. Free ATTO647 was 110-fold brighter. This factor also marks the threshold for the maximum increase of fluorescence signal that can be expected upon complete unfolding of MBs in bulk experiments of our study. Unfolding of the MBs by denaturation at pH 11, showed a 25-fold increase in fluorescence signal. At the same pH, the signal of free ATTO647 was four-fold higher than that of the beacon, indicating incomplete denaturation of the beacon. Next, we measured the fluorescence signal of the MB in the presence of excess binding sites that were provided as synthetic, custom-designed RNA oligonucleotides. The oligonucleotides encoded two MB binding sites separated by a spacer (2xMB RNA). In the presence of 1 µM 2xMB RNA—a 4-fold excess of MB targets—the fluorescence signal of the MBs increased 50-fold. With a 20-fold excess of targets, the MB signal was 54-fold higher. The results confirm a substantial increase in fluorescence of MBs as they unfold and bind their target sequence. These results also suggest that with MBs in solution, it should be possible to visualize individual transcripts as distinct bright diffusive spots.

To verify MB specificity, IVT reactions were performed with either 10 pM 32xBT DNA or 10 pM Spi32 DNA – encoding 32 Spinach aptamer repeats – as template in the presence of 500 nM MB. No change in fluorescence was observed in the reaction with Spi32 DNA as the template (Fig. 1c, green). The normalized fluorescence signal of the MB + Spi32 DNA reaction was comparable to the ‘MB only’ negative control sample prepared with transcription mix, T7 RNAP, 500 nM MB, but no DNA (Fig. 1c, black). Conversely, the transcription reaction with 32xBT DNA as template exhibited a 4.5- to 5.3-fold increase in the fluorescence signal (Fig. 1c, red). And, numerous bright diffusive spots were observed in the ‘MB + 32xBT’ reaction but not in the ‘MB + Spi32’ or ‘MB only’ reactions. To ensure that our observations of the ‘MB + Spi32’ reaction was not merely due to a failure of transcription, we confirmed that both, the Spi32 DNA and 32xBT DNA templates were transcribed by resolving the denatured RNA reaction products on gel (Supplementary Fig. S2). Thus, we established the sequence specificity of the MBs and even observed mRNA molecules produced from the DNA template.

Next, we compared MB-based smIVT to the traditional Spinach aptamer-DHFBI approach for visualizing mRNA^25^(Supplementary Fig. S3). The Spinach aptamer is an 84-base RNA sequence that folds into a G-quadruplex. The folded structure stabilizes a molecule of DHFBI—a fluorophore that resembles the chromophore of GFP—and enhances its fluorescent properties^26^. While Spinach has been successfully applied for mRNA measurements by others^14,15^, we could not demonstrate its utility for single-molecule mRNA detection. Visualization of in vitro transcription reactions with Spi32 DNA template in the presence of the Spinach aptamer-binding DHFBI dye did not show bright spots of diffusing mRNA molecules; only a few, faint spots barely detectable above the noise (Supplementary Fig. S3). This makes for a drastic contrast between molecular beacons and Spinach-DHFBI. We expect that the poor performance of the Spinach aptamer results from incomplete folding owing to its length (84 bases), and its misfolding due to self-complementarity in the aptamer sequence^27–29^. The latter is corroborated by RNA structure prediction tools^27–29^ (Supplementary Fig. S3).

Comparing the performance of commercial kit-sourced T7 RNA polymerases.

Having established the sequence-specificity and bimodal fluorescent properties of MBs, and the suitability of MBs as probes for smIVT, we applied smIVT to compare the performance of commercially available T7 RNAP-based IVT kits. In addition to being used for in vitro transcription, IVT kits may be incorporated into home-made cell-free expression systems that can be enclosed with template DNA in GUVs to mimic synthetic cells. The applicability of IVT kits in synthetic cell research makes it non-trivial to identify the most suitable kits for DNA concentrations in the pM range. Eight commonly used T7 RNAP kits (Table 1) were selected based on their prevalence in impactful research papers on in vitro transcription^30^. We have tabulated the composition of the kits in as much detail as possible (Table 2), given that some manufacturers chose not to disclose the composition of kit components. We have also summarized the different definitions of activity units for T7 RNAP, which differ among manufacturers (Table 2).

**Table 1:** List of product names, manufacturers, and catalogue numbers of the commercial T7 RNA polymerase kits used in this study.

T7 RNAP kit	Product name	Manufacturer	Catalogue number
NEB	T7 RNA Polymerase	New England Biolabs, USA	M0251S
NEB HiScribe	HiScribe^®^ T7 Quick High Yield RNA Synthesis Kit	New England Biolabs, USA	E2050S
Invitrogen	T7 RNA Polymerase	Thermo Fisher Scientific, USA	18,033,019
Promega	T7 RNA Polymerase	Promega, USA	P2075
Roche	T7 RNA Polymerase	Roche, CH	RPOLT7-RO
Takara	T7 RNA Polymerase ver2.0	Takara Bio, JP	2541 A
Thermo Fisher	T7 RNA Polymerase	Thermo Fisher Scientific, USA	EP0111
TranscriptAid	TranscriptAid T7 High Yield Transcription Kit	Thermo Fisher Scientific, USA	K0441

**Table 2:** Summary of the composition of the 1X IVT reaction buffer, composition of the T7 RNAP storage buffer, and the definition of an enzymatic unit of the commercial T7 RNAP kits used in this study as disclosed by the manufacturer.

T7 RNAP kit	Reaction buffer from the kit diluted to 1X	T7 RNAP storage buffer	Unit definition
NEB (#M0251S)	40 mM Tris-HCl 6 mM MgCl_2_ 1 mM DTT 2 mM spermidine	50 mM Tris-HCl 100 mM NaCl 20 mM β-ME 1 mM EDTA 50% Glycerol 0.1% (w/v) Triton^®^ X-100 pH 7.9 at 25 °C	“One unit is defined as the amount of enzyme that will incorporate 1 nmol ATP into acid-insoluble material in a total reaction volume of 50 µl in 1 h at 37 °C.” Reaction conditions: 40 mM Tris-HCl, 6 mM MgCl_2_, 1 mM DTT, 2 mM spermidine.
NEB HiScribe (#E2050S)	Not disclosed	Not disclosed	Not disclosed
Invitrogen (#18033019)	40 mM Tris-HCl; pH 8.0 10 mM MgCl_2_ 2.5 mM spermidine-(HCl)_3_ 25 mM NaCl	20 mM potassium phosphate buffer; pH 7.7 1 mM EDTA 10 mM DTT 100 mM NaCl 0.1% Triton^®^ X-100 50% (v/v) glycerol.	“One unit hydrolyzes 1 nM of ribonucleotide into acid-precipitable material in 1 h at 37 °C using a T7 transcription vector as template.” Reaction conditions: 40mM Tris-HCl (pH 8.0), 25 mM NaCl, 8 mM MgCl_2_, 2 mM spermidine-(HCl)_3_, 5 mM DTT, 0.4 mM ATP, 0.4 mM CTP, 0.4 mM GTP, 0.4 mM UTP, 1 µCi [3 H]GTP, 1 µg SphI-cut pT7L13, and enzyme in 50 µL for 10 min. at 37 °C
Promega (#P2075)	40mM Tris-HCl; pH 7.9 6 mM MgCl_2_ 2 mM spermidine 10 mM NaCl	20 mM potassium phosphate buffer; pH 7.7 at 25 °C 1 mM EDTA 10 mM DTT 100 mM NaCI 0.1% (v/v) Triton^®^ X-100 50% (v/v) glycerol	“One unit is defined as the amount of enzyme required to catalyze the incorporation of 5 nmol of CTP into acid-insoluble product in 60 min at 37 °C in a total volume of 100 µl.” The reaction conditions were: 40 mM Tris-HCl (pH 7.9 at 25 °C), 6 mM MgCl_2_, 10 mM DTT, 10 mM NaCl_2_, 2 mM spermidine, 0.05% Tween^®^ 20, 0.5 mM each of ATP, GTP, CTP, and UTP, 0.5 µCi [3 H]CTP and 2 µg supercoiled pGEM^®^-5Zf(+) Vector DNA.
Roche (#RPOLT7-RO)	40 mM Tris-HCl; pH 8.0 at 20 °C 6 mM MgCl_2_ 10 mM DTT 2 mM spermidine	10 mM potassium phosphate 200 mM KCl 0.1 mM EDTA 30 mM β-ME 50% glycerol (v/v) 0.1% Tween 20 pH 7.9 at 4 °C	“One unit is the enzyme activity which incorporates 1 nmol CMP in acid-precipitable RNA products within 60 min at + 37 °C.” Reaction conditions: 40 mM Tris-HCl pH 8.0 at 20 °C, 6 mM MgCl_2_, 10 mM DTT, 2 mM spermidine, pH approximately 8.0 at 20 °C.
Takara (#2541A)	Not disclosed	Not disclosed	“One unit is defined as the amount of enzyme that incorporates 1 nmol of [3 H]GMP into the acid-insoluble fraction in 1 h at 37℃.” Reaction conditions: 40 mM Tris-HCl pH 8.0, 8 mM MgCl_2_, 2 mM spermidine, 5 mM DTT, 0.4 mM ATP, UTP, and CTP, 0.4 mM [3 H]GTP, 1 µg/50 µl pT7-2 DNA
Thermo Fisher (#EP0111)	40 mM Tris-HCl; pH 7.9 at 25 °C 6 mM MgCl_2_ 10 mM DTT 10 mM NaCl 2 mM spermidine	25 mM HEPES-NaOH; pH 7.6 0.1 mM EDTA-Na_2_ 150 mM NaCl 10 mM DTT 0.09% Triton^®^ X-100 50% glycerol	“One unit of the enzyme incorporates 1 nmol of AMP into a polynucleotide fraction in 60 minutes at 37°C.” No reaction conditions explicitly provided.
TranscriptAid (#K0441)	Not disclosed	Not disclosed	Not defined

**Fig. 2:**
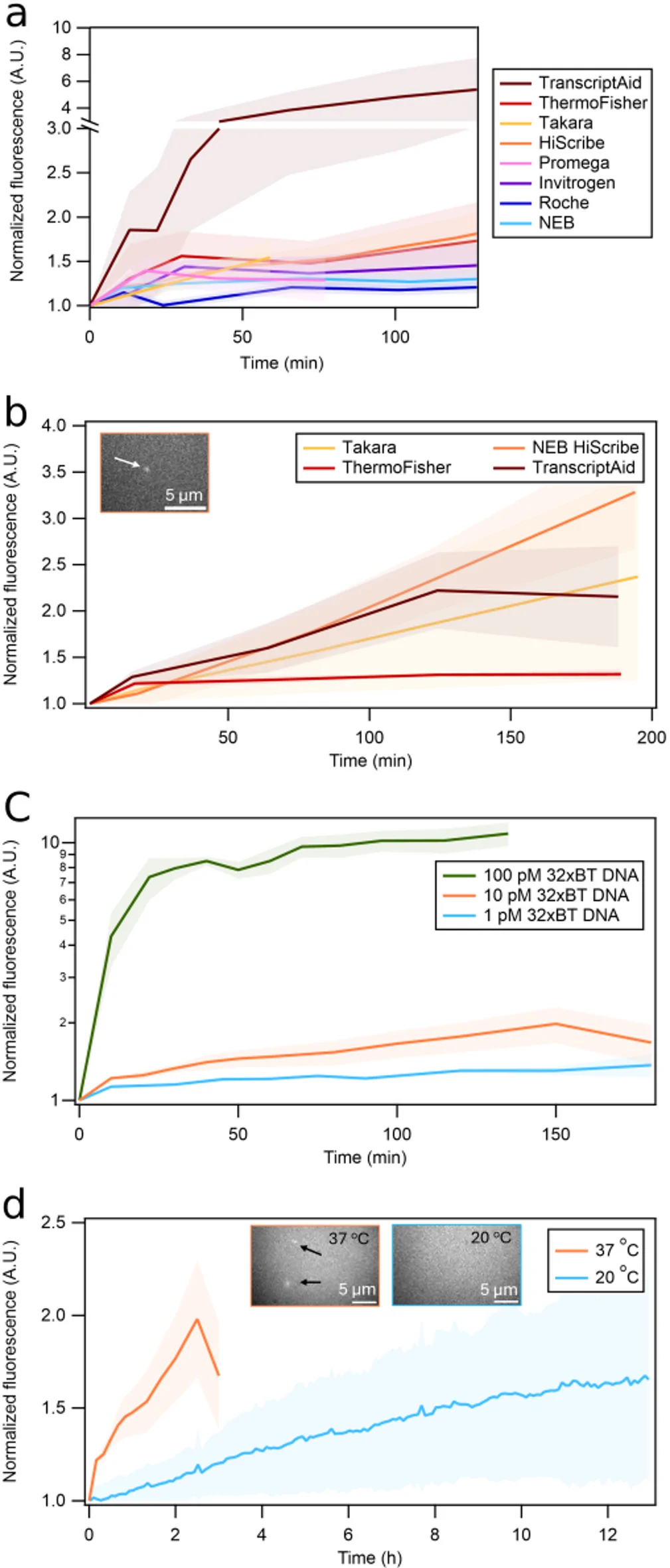
A comparison of the performance of commercially-available IVT kits for template concentrations in the low pM range. Performance of kits in (**a**) the buffer provided by the manufacturer, and (**b**) home-made PURE-mimicking buffer. The impact of (**c**) template concentration and (**d**) reaction temperature on the performance of NEB HiScribe.

To compare the performance of the kits, reaction mixes for each kit (*N* = 3) were prepared according to the manufacturer’s instructions with 10 pM 32xBT DNA as template, and 500 nM MB as the mRNA probe. The reactions were incubated at 37 °C. Aliquots of the reactions were taken at regular intervals and the fluorescence signal was measured at room temperature (20 °C). Since the fluorescence of a dye is affected by its buffer composition, and we use a fluorescence-based probe to compare the performance of eight kits each with a different buffer, fluorescence signals were normalized to enable comparison. For this, the background-corrected fluorescence signal of each measurement was normalized to the background-corrected fluorescence signal of the IVT preparation with 500 nM MB before the start of transcription, t = 0. The fluorescence signal at this time point was assigned an arbitrary value of 1. Importantly, the fluorescence signal at t = 0 was not merely noise, but the strong background signal of 500 nM MB that was 10-fold higher than the noise. Considering that the brightness of each unfolded MB is expected to be – as measured in bulk experiments – approximately 110-fold higher than its folded state (Fig. 1b), increase in normalized fluorescence can be treated as proportional to mRNA concentration in solution (Fig. 2a).

The results show that TranscriptAid (Thermo Fisher Scientific, USA) was the best performing IVT kit for template concentrations in the pM range. The fluorescence signal increased over 5-fold in 2 h (Fig. 2a). NEB HiScribe (New England Biolabs, USA), Thermo Fisher (Thermo Fisher Scientific, USA), and Takara (Takara Bio, Japan) IVT kits exhibited fluorescence signals between 1.5- to 2-fold higher than at t = 0 after 2 h (Fig. 2a). Invitrogen (Thermo Fisher Scientific, USA), Roche (Roche, Switzerland), NEB (New England Biolabs, USA), and Promega (Promega, USA) IVT kits had the lowest performance with a fluorescence fold change of less than 1.5 (Fig. 2a). The four highest performing IVT kits were chosen to evaluate their performance the reaction buffer of the PURE cell-free expression system^9^.

The PURE system is a cell-free, in vitro transcription-translation system comprising a defined (and customizable) mix of recombinant enzymes, tRNAs, and purified ribosomes that is widely used in synthetic biology^9^. Owing to its defined composition, PURE has found substantial value in the development of synthetic cells. The PURE system buffer is optimized to support the performance of T7 RNAP and *E. coli*-sourced translation machinery. We evaluated the four best-performing IVT kits (see above) for their functionality in home-made PURE mimicking buffer^8^. We define the buffer we use as a PURE mimicking buffer since it lacks creatine phosphate required for energy regeneration. This set of measurements minimizes kit-to-kit differences, limiting them to variations in the T7 RNAP storage buffers (Table 2), and, potentially, the T7 RNAP mutant used in the kit. Here, TranscriptAid performed similarly to the other kits (Fig. 2b). NEB HiScribe was the best-performing kit. It produced comparable results under both native and PURE buffer conditions, had the highest and most consistent fluorescence increase in the PURE buffer, and was characterized by the emergence of bright diffusive mRNA-MB complexes. Hence, it was chosen as the T7 RNAP kit for further experiments.

Template copy number modulates the transcription reaction rate. The fluorescence signal of the IVT reaction for NEB HiScribe T7 RNAP with 1 pM, 10 pM, and 100 pM 32xBT DNA template concentrations was measured with 500 nM MB to compare how DNA template concentration affects reaction output over time (Fig. 2c). Indeed, IVT with 100 pM template has the highest output followed by 10 pM, and 1 pM, with mRNA output decreasing non-linearly with template concentration. Furthermore, while the fluorescence signal for the reactions with 1 pM and 10 pM template concentrations increases gradually over 3 h, 70% of the increase in the fluorescence signal for the reaction with 100 pM template occurs in the first 30 min after which the signal increases only minimally. Since the NEB HiScribe kit is expected to produce ~ 5–10 µg of mRNA in 2 h for 20 ng of DNA template (100 pM of 32xBT DNA in a 50 µL reaction), the slow increase in fluorescence signal after 30 min is unlikely due to exhaustion of ribonucleotides or ATP in the reaction. It is possible that under optimized buffer conditions, T7 RNAP transcribed mRNA, and effectively, MB targets rapidly enough to deplete MB in solution^31^. SmIVT experiments performed using higher DNA concentrations may require proportionally higher MB concentrations.

### Effects of temperature on smIVT

T7 RNA polymerase functions optimally at 37 °C, with transcription rates decreasing when the temperature is lowered to 20 °C—a temperature that offers the convenience of working outside an incubator. At both temperatures in PURE, transcription by T7 RNAP proceeds faster than translation by the *E. coli*-derived machinery, and therefore, is not limiting^15^. Thus, we explored whether the cost of slower T7 RNAP activity and lower yields is justified by the convenience of working smIVT at room temperature. We compared the performance of our assay at 20 °C versus 37 °C.

The reaction rates and fluorescence were remarkably different between 20 °C and 37 °C. While the fluorescence signal reached its maximum within 2.5 h at 37 °C, it took 10 h at 20 °C for the fluorescence signal to reach half that value (Fig. 2d). However, the reaction sustained for a long period with the gradual increase in fluorescence signal observed even after 80 h (Supplementary Fig. S4). Interestingly, at 37 °C we observed numerous diffusive fluorescent spots, which became more abundant over time. In contrast, the signal remained homogeneous over the field of view at 20 °C for the entire duration of the measurement (Fig. 2d).

The increase in fluorescence, but absence of distinct spots at 20 °C in our observations cannot be explained by degradation of the MBs separating the fluorophore from the quencher – enzymatic degradation by RNase is unlikely since we use a methylated molecular beacon. Even so, all experiments are performed with RNase-free components. Our use of quality-controlled commercially supplied kits and buffered solutions rules out chemical degradation. An increase in background fluorescence overtime as a result of melting of the molecular beacons is also unlikely since the measurement was consistently performed at room temperature. We expect that the homogeneous increase in fluorescence and absence of fluorescent spots in reactions performed at 20 °C is due to the poor accessibility of MBs for their target sites at that temperature. Both, MBs and their target sites on the nascent mRNA, fold into hairpins and exist in an equilibrium between the closed and open states. Lower temperatures shift the equilibrium towards the closed state, effectively decreasing the proportion of MBs conducive for binding, and the proportion of accessible MB target sequences^19^.

### Encapsulated transcription

The PURE cell-free expression system can be enclosed with template DNA in GUVs to mimic synthetic cells. To evaluate whether smIVT can detect mRNA transcripts in such a set-up, we first prepared a transcription reaction—comprising NEB HiScribe transcription mix, 10 pM template DNA, 500 nM MB, and 0.4X SYBR Gold intercalator (Thermo Fisher Scientific)—and then encapsulated the solution within GUVs. The GUVs were visualized immediately after preparation, and at two additional time points. GUVs were incubated at 37 °C but imaged at room temperature (20 °C). During microscopy, imaging was biased towards fluorescent GUVs.

**Fig. 3:**
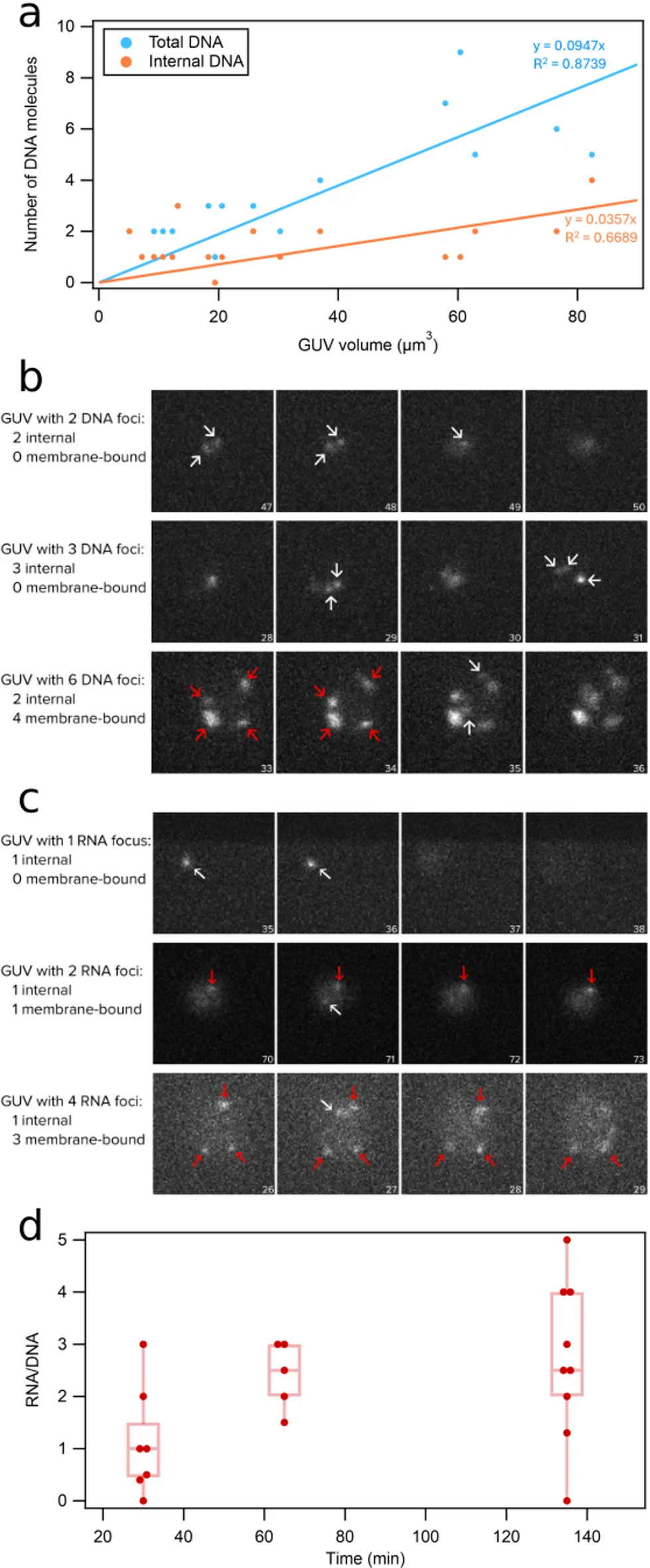
SmIVT in GUVs. (**a**) The number of DNA molecules encapsulated in GUVs of varying sizes follows a linear fit. The DNA can also adhere to the membrane, hence, total DNA in the GUV (blue) is separated between the membrane-bound and the freely-diffusing internal DNA (orange). (**b**) Representative images of GUVs with internal (white arrows) and membrane-bound (red arrows) DNA; SYBR Gold labelling. (**c**) Representative images of GUVs with internal (white arrows) and membrane-bound (red arrows) RNA; MB labelling. (**d**) As transcription progresses within GUVs, the RNA/DNA ratio increases.

A DNA concentration of 10 pM, corresponds to 0.5 DNA molecules in a GUV with a diameter of 5 μm (volume: ~ 65 µm^3^). We compared this to the total count of SYBR Gold-stained DNA molecules inside GUVs that we observed experimentally. The data show a linear increase of DNA copies with GUV size. However, the linear fit indicates that the template concentration within GUVs is 123 ± 16 pM (Fig. 3a, blue), 12 times higher than the transcription reaction prepared in bulk for encapsulation. This shows large encapsulation heterogeneity and, hence, GUV-to-GUV variability of in vitro transcription. The heterogeneity underlines the necessity for visualizing transcription at a single-molecule level, at the scale of individual GUVs.

A closer look at the fluorescence data revealed two distinct populations of DNA molecules in GUVs: molecules freely diffusing in the GUV interior (Fig. 2b, white arrows), and membrane-bound molecules (Fig. 2b, red arrows), probably adhered to defects on the membrane, such as pockets. Membrane defects arise when stable bilayer formation is affected. They are, typically, more common in GUVs encapsulating complex solutions such as a macromolecular mix of the building blocks for synthetic cells. Notably, in our hands, GUV yield was lower when encapsulating the transcription mix, compared to only buffer.

Of the two DNA populations, only the freely diffusing molecules are of substantial interest for our assay, as they are individually distinguishable and fully accessible to the transcription machinery. Thus, we adjusted the DNA counts, considering only the molecules in GUV interiors (Fig. 3a, orange). We calculated the effective DNA concentration to be 46.4 ± 15.4 pM – ~2.5-fold lower than the concentration of total DNA in GUVs. These numbers emphasize a considerable loss of template DNA to the membrane and that, effectively, the stochastic concentration effect – where some GUVs have more DNA molecules than expected—is amplified.

The mRNA molecules in GUVs were also distributed in two populations: mRNAs freely diffusing in the GUV interior (Fig. 3c, white arrows), and mRNAs bound to the membrane (Fig. 3c, red arrows). Some membrane-associated clusters were much larger and brighter than the individual diffusive mRNA molecules, suggesting the presence of multiple mRNA copies at these foci.

To verify that transcription proceeded in GUVs, and to assess it quantitatively, we determined RNA and DNA counts in GUVs at 30 min, 65 min, and 2 h 15 min. The time point of 30 min corresponds to 30 min after the transcription reaction was prepared, and to the time point immediately after GUV preparation. The protocol for enclosing the transcription reaction in GUVs with the inverted emulsion method takes 30 min. We plotted the RNA/DNA for the three points (Fig. 3d) and determined that after 2 h 15 min, there were, on average, three mRNA molecules produced from a single DNA. This confirms that smIVT can be successfully used to track in vitro transcription at single-molecule resolution over time.

## Discussion

Building on earlier bulk measurements of cell-free expression systems^14,15^, we showcase the benefits of using molecular beacons as probes for studying transcription at single molecule resolution, both, in solution and in GUVs. We have demonstrated how to visualize transcription in GUVs that carry < 10 DNA copies—an order of magnitude comparable to natural cells where only one or two DNA copies are typically present. Molecular beacon-based smIVT is a powerful technique for quantitative, bottom-up synthetic biology studies. It can be employed to improve our understanding of transcription in proto-synthetic cells; for instance, to reveal the effect of physicochemical conditions like macromolecular crowding on transcription kinetics, the impact and role of stochastic noise in transcription, and transcription-translation coupling.

Nevertheless, MB-based smIVT is not without its limitations. MB-labelled mRNA foci differed in brightness, which could not be accounted for by the illumination profile of the field of view or by differences in z-position of the foci. This indicates a variation in the number of MBs that bind to target mRNAs. Resolving the denatured transcription products on an agarose gel showed that all the transcripts were longer than the expected length of 1.6 kb (Supplementary Fig. S2). This may be a consequence of using T7 RNAP as the transcription enzyme. T7 RNAP is an incredibly efficient macromolecular machine. It is a single-protein enzyme recognizing a highly specific promoter (consensus sequence: 5’-TAATACGACTCACTATAG-3’) that polymerizes NTPs at a rate of > 200 nt/s under optimal conditions^32^. Non-ideally, however, T7 RNAP does not always require its promoter to initiate transcription. T7 RNAP can transcribe a dsDNA template carrying a 3’ overhang, and on rare instances (< 1%) also blunt-ended DNA or DNA with a 5’ overhang^33^. While we used PciI restriction enzyme that generates 5’ overhangs for linearizing pET-32xBT, and filled-in the overhangs of the linearized template using Klenow, the brightest band on the gel at ~ 4.5 kb matches the distance from the 5’ end of the plus strand of the template to the T7 terminator, located at position 4483. The band at running slightly heavier than 6 kb is comparable to the length of the linearized DNA at 6696 bp. T7 RNAP is capable of product-templated transcription when the 3’ ends of its transcripts fold into a hairpin (loopback transcription), or when the RNA template hybridizes to another nucleic acid chain providing an exposed 3’-OH^34^. This may explain the presence of heavier transcripts. The diversity of transcripts may partly account for the difference in brightness of the mRNA foci—longer transcripts, especially loopback transcripts, would carry more MB target sites and consequently, be brighter. Employing T7 RNAP_G47A,884G_ that reduces dsRNA byproducts of T7RNAP transcription is advised^35^.

We observed a range of activity among the IVT kits that we tested (Fig. 2a), but it is not immediately clear what causes this variation. Considering only those kits with disclosed reaction components and buffer compositions (NEB, Invitrogen, Promega, Roche, and Thermo Fisher), the kits use the same T7 RNAP variant, include 2–2.5 mM spermidine in the reaction buffer to enhance transcription, and are buffered with Tris-HCl at a pH of 7.9 or 8. The kits differ in the use of reducing agents like dithiothreitol (DTT) and β-mercaptoethanol (β-ME). DTT and β-ME reduce disulfide bonds (–S–S–) in proteins and protect free thiol (–SH) moieties. This prevents protein aggregation and maintains enzymatic activity. Notably, the kits also differ in the MgCl_2_ content of the buffers, and in the nucleotide concentration recommended for use. MgCl_2_ and nucleotide concentration affects IVT reaction rates. Increasing nucleotide concentration speeds up transcription as long as it does not exceed the MgCl_2_ concentration. Otherwise, T7 RNAP activity is impeded. Thus, the optimal concentration of MgCl_2_ is slightly above the (total) nucleotide concentration^36^. The IVT kits also differ in the concentration of T7 RNAP (in units per volume), and in the definition of the enzymatic unit. For instance, Thermo Fisher defines 1 unit of T7 RNAP as the enzyme required to incorporate 1 nmol of AMP into RNA at 37 °C in 1 h. NEB defines it similarly, and establishes that the reaction occurs in 50 µL. Thermo Fisher and Roche also share similar definitions, but while Thermo Fisher follows AMP, Roche follows CMP. Additionally, the buffer conditions under which the unit is defined differs between kits. Thus, the unit concentrations of the enzymes are not directly comparable and highlight the need for industry-wide standards. We conclude that the variation in the performances of the kits we tested stem from differences in T7 RNAP, nucleotide, and MgCl_2_ concentrations.

While it is tempting to use the highest-yield transcription system for a synthetic cell, it is essential to ensure compatibility of different systems. For example, *E. coli*-based translation in PURE falls behind the rapid T7-based transcription^15^. Artificially slowing the transcription to efficiently couple transcription and translation would be beneficial for efficient energy use in a synthetic cell. The smIVT assay reported here can be applied to study transcription with real-time single-molecule resolution. Coupled with real-time translation tracking, smIVT can better our understanding of how to reconstitute and couple transcription-translation in synthetic cells.

## Methods

### 32xBT construct

The pET-32xBT plasmid was produced as previously described^37^. The plasmid was digested with *PciI* (New England Biolabs)—a single-cutter in pET-32xBT—and ends were filled with DNA Polymerase I, Large (Klenow) Fragment (New England Biolabs) .

### Transcription reaction

With the exception of experiments performed with the PURE buffer, IVT reactions were performed according to the manufacturer’s instructions with 500 nM MB, and with non-standard choices made for template concentration (1, 10, or 100 pM), and in some tests for temperature (37–20 °C). Transcription mixes for IVT reactions performed in PURE buffer were made with 10 pM 32xBT DNA, 500 nM MB, 5 mM NTPs (New England Biolabs), 5 mM DTT (New England Biolabs), and 1 U/µL RNase inhibitor (2313 A, Takara Bio) in a PURE system-mimicking buffer containing 20 mM HEPES pH 7.5, 150 mM potassium glutamate, 13 mM magnesium acetate, 1% BSA, 2 mM spermidine, 2 mM NaN_3_. For encapsulated transcription studies, SYBR Gold dye (Thermo Fisher Scientific) diluted to 0.4x of its commercially-advised working concentration was added to IVT reactions to image DNA.

For continuous measurement at room temperature, 20 µL of the reaction solution was sealed in a chamber and placed in a microscope.

### GUV production

Giant unilamellar vesicles were produced using the inverted emulsion method^21^, which was optimized for encapsulating a complex solution. 50 µL of 0.4 mg/mL DOPC in 65% glyceryl trioleate (T7752, Sigma-Aldrich) was distributed on top of 40 µL of an aqueous solution comprising 185 mM glucose, 19 mM MgCl_2_, and 40 mM Tris-HCl pH 7.6 in a 1.5 mL tube. The preparation was incubated at room temperature for at least 4 h to form a stable lipid monolayer on the oil-water interface. In a separate tube, 150 µL of 0.4 mg/mL DOPC in 65% glyceryl trioleate was warmed to 40 °C on a heating block. 2.0 µL of the IVT reaction with 200 mM was mixed into the warmed oil. To produce minuscule water-in-oil droplets, this tube was rubbed against a tube rack until the solution turned opaque. The resulting solution was pipetted onto the oil-on-water phase-separated solution prepared earlier. The tube was centrifuged at 300x*g* for 20 min. The oil phase was carefully pipetted off, and a 20 µL aliquot of the aqueous phase containing GUVs was used for measurements.

### RNA electrophoresis in agarose gel

2x RNA loading dye (B0363S, New England Biolabs) was added to the transcription reaction in a 1:1 ratio. The sample was denatured at 70 °C for 10 min and then resolved on a 1% agarose gel.

### Fluorescent imaging setup

The measurements were performed on a custom-built inverted microscope (based on Nikon Eclipse TE2000-U) that combines bright-field and wide-field fluorescent imaging^38^. Water-immersion objective Nikon Plan Apo x 60 NA = 1.2 was used for most measurements. As water on an objective evaporates in about 12 h, an immersion oil with a refraction index matching that of water (*n* = 1.334) was used for longer experiments. All samples were measured in a sealed chamber (GeneFrame, Thermo Fisher Scientific) to prevent evaporation.

The following combinations of excitation lasers and filters were used: 639 nm 40mW CW laser (Coherent) and FF01-698/70 bandpass filter (Semrock Inc.) for ATTO647N-functionalized molecular beacon; 532 nm 50 mW CW laser (Cobolt Samba) and FF01-580/60 bandpass filter (Semrock Inc.) for Spinach aptamer with DFHBI-1T dye; 491 nm 50 mW CW laser (Cobolt Calypso) and FF03-525/50 bandpass filter (Semrock Inc.) for SYBR Gold dsDNA intercalator dye.

All fluorescent imaging was performed on an EMCCD camera (iXON+ 897E, Andor Technology), controlled via MicroManager. Bright-field imaging was performed with 450 nm diode (Roithner Lasertechnik GmbH) illumination and CMOS camera (DCC1545M, Thorlabs) detection.

Custom-written Python scripts were developed specifically for image analysis of the bulk measurements. The center of the illumination Gaussian in the field of view for the first frame was determined and the mean signal in a 25-pixel radius for all consequent frames was plotted. The background value was obtained from a separate fluorophore-free water measurement and subtracted from all stacks.

### DNA and RNA quantification in GUVs

Fluorescence microscopy videos were examined frame-by-frame and the number of fluorescent DNA and RNA foci were counted manually. Foci that freely diffused in and out of focus within the GUV were classified as internal while foci that remained associated with the boundary of the GUV were classified as membrane-associated.

### Nucleotide sequences

Molecular beacon (MB-32xpET-A647N-IBRQ):5’-/5ATTO647NN/mCmCmGmCmAmAmAmUmAmAmAmUmUmUmAmAmGmGmGmUmAmAmGmCmGmG/3IAbRQSp/-3‘.

*Complementary RNA*: 2xMB RNA5‘-rCrUrUrArCrCrCrUrUrArArArUrUrUrArUrUrUrGrCrArUrArUrArUrArUrCrUrUrArCrCrCrUrUrArArArUrUrUrArUrUrUrGrC − 3‘.

### T7 RNA polymerase kits

Product names, manufacturers, and catalogue numbers of the commercial T7 RNA polymerase kits used in this study are provided in Table 1. The composition of the reaction buffers, and T7 RNAP storage buffers as disclosed by the manufacturers are summarized in Table 2. The definition of an enzymatic unit for each kit is also included.

## Supplementary Information

Below is the link to the electronic supplementary material.

Supplementary Material 1

## Data Availability

The datasets generated and analyzed for this study are available in the DataverseNL repository, 0.34894/I4WR8M (10.34894/I4WR8M). Custom-written Python scripts developed for image analysis are available on Github (https://github.com/bvadg1002/smIVTT).
