# Supplementary material for: Employing molecular beacons to assess in vitro transcription with single-molecule resolution
Source: Sci Rep. 2026 Jun 10;16:26405. doi: 10.1038/s41598-026-55220-6 (PMC13500459; doi:10.1038/s41598-026-55220-6)

Supplementary Information

Employing molecular beacons to assess *in vitro* transcription with single-molecule resolution

Vadim G. Bogatyr[1†], Fatema-Zahra M. Rashid[1†], Andreas S. Biebricher[1], Wilhelm T.S. Huck[2], Gijs J.L. Wuite[1*]

[1] Department of Physics and Astronomy, Physics of Living Systems, VU University Amsterdam, De Boelelaan 1100, Amsterdam 1081HV, The Netherlands

[2] Institute for Molecules and Materials, Radboud University, Heyendaalseweg 135, 6525 AJ Nijmegen, The Netherlands

[†] These authors contributed equally to this work

* Corresponding author: g.j.l.wuite@vu.nl

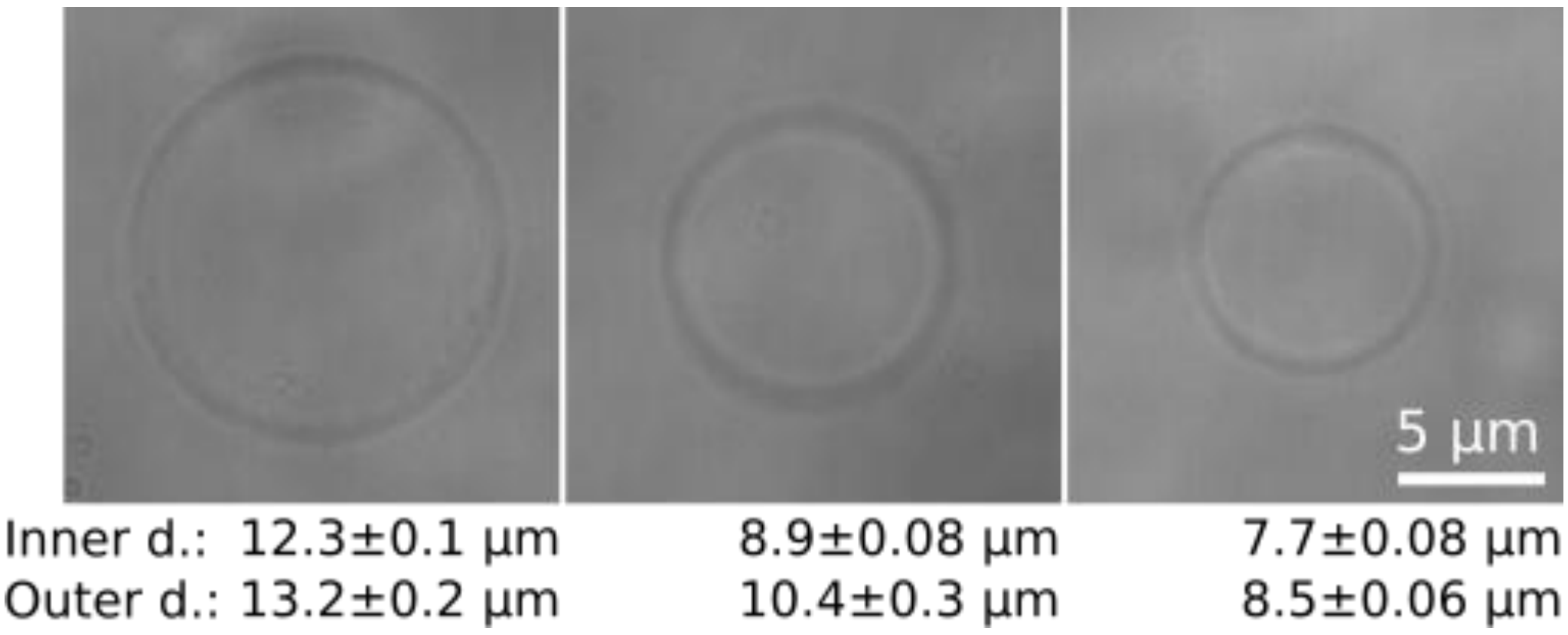

Figure S1: Typical GUVs obtained using the inverted emulsion method.

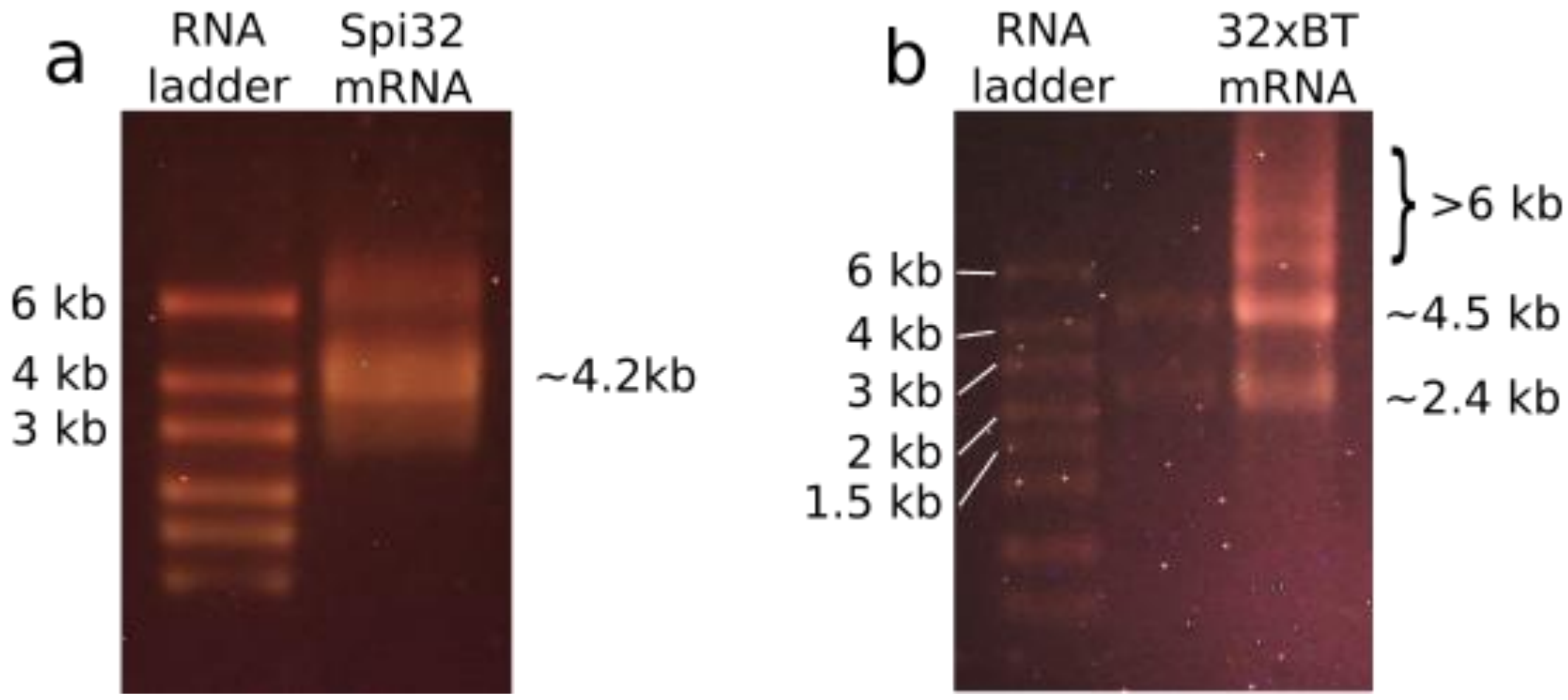

Figure S2: Denaturation agarose gels of IVT reactions with **(a)** Spi32 DNA and **(b)** 32xBT DNA as template show that both templates were transcribed. **(a)** Transcripts from the Spi32 DNA template were of the expected length of 4.2 kb. **(b)** RNA molecules of several discrete lengths were transcribed from the 32xBT DNA, all of which were longer than the expected size of 1.6 kb (see Discussion).

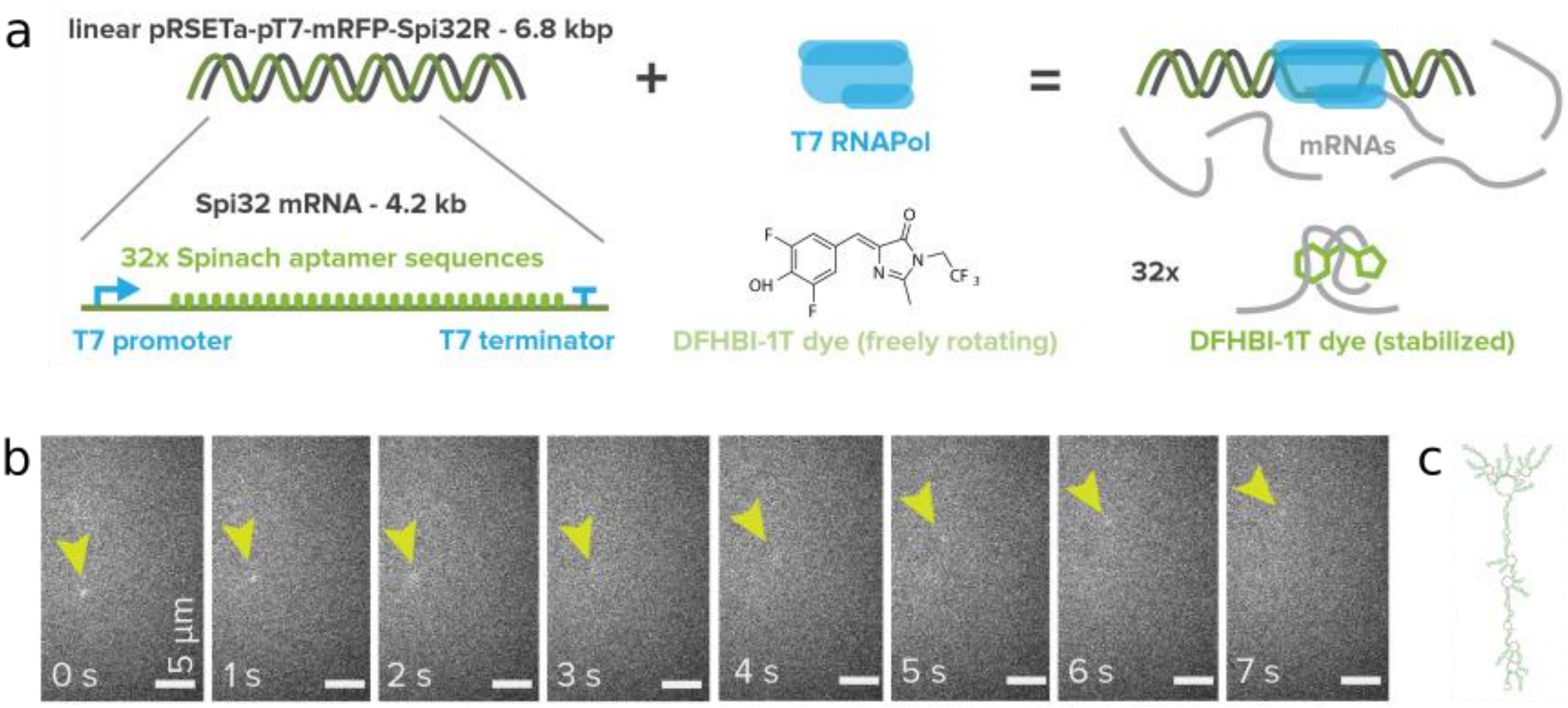

Figure S3: Visualizing mRNA with Spinach aptamer and DHFBI-1T. **(a)** Linearized pRSETa-pT7-mRFP-Spi32R plasmid encodes a gene carrying 32 Spinach aptamer repeats (Spi32) flanked by a T7 promoter and terminator. When transcribed, the aptamers of the Spi32 mRNA fold into G-quadruplexes that stabilize the DFHBI-1T dye, enhancing its fluorescent properties. **(b)** In vitro labelling of Spi32 mRNA with DHFBI-1T did not yield bright spots of diffusing mRNA. Only a few faint spots barely detectable above the background signal were observed. Reaction conditions: NEB HiScribe kit, 10 pM linearized pRSETa-pT7-mRFP-SPi32R (Spi32 DNA), 4 µM DFHBI-1T, 37 °C for 2 h. **(c)** The modeling of an 8x Spinach repeat RNA sequence (instead of 32 due to computational limitations) indicated that the RNA can fold onto itself due to multiple sequence repeats. This disrupts G-quadruplex assembly.

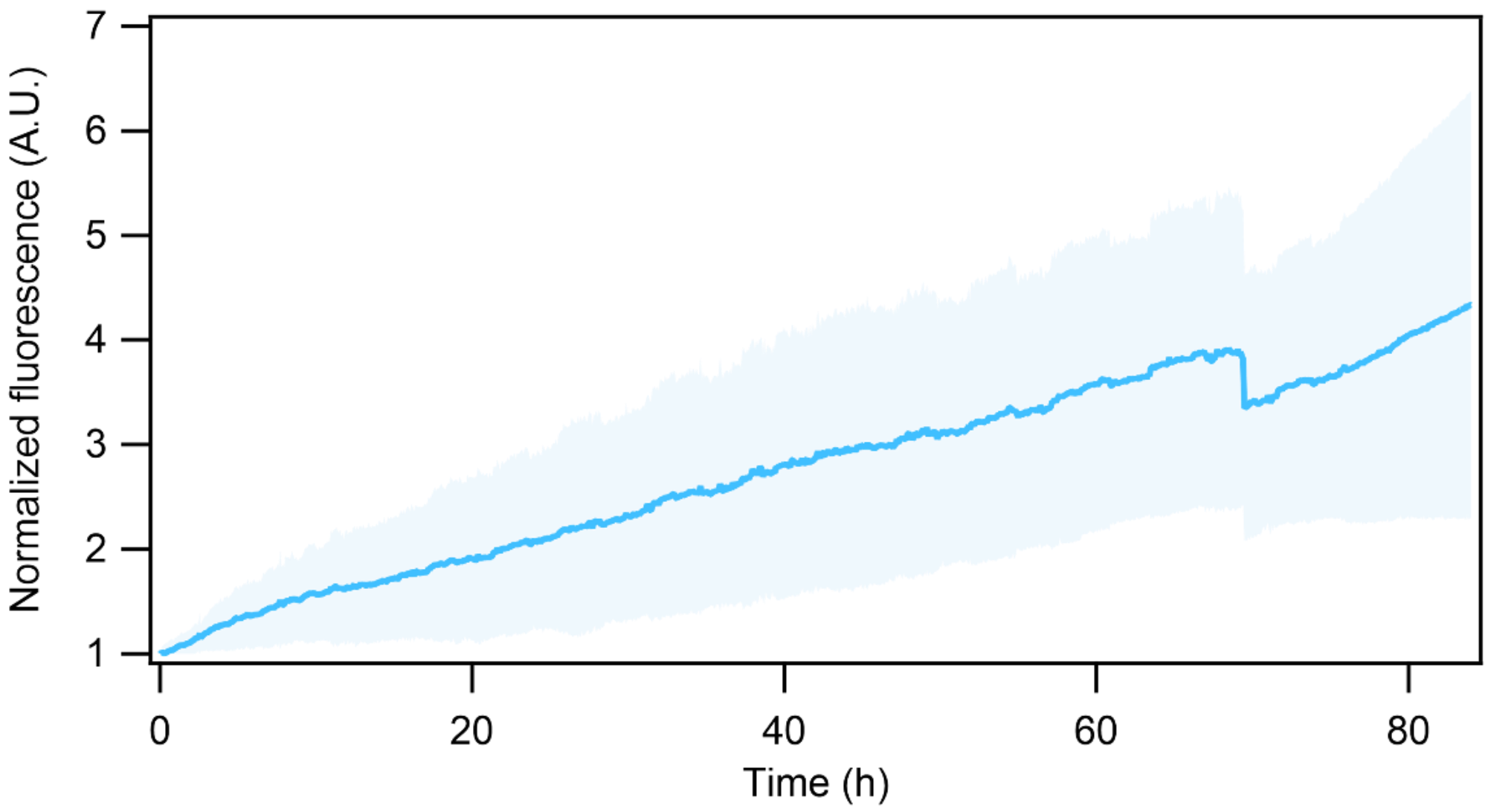

Figure S4: In vitro transcription of 32xBT DNA at 20 °C with the NEB HiScribe kit (New England Biolabs) sustained for at least 84 h at which point the measurement was stopped.

Uncropped and unedited agarose gel images

Figure S2a:

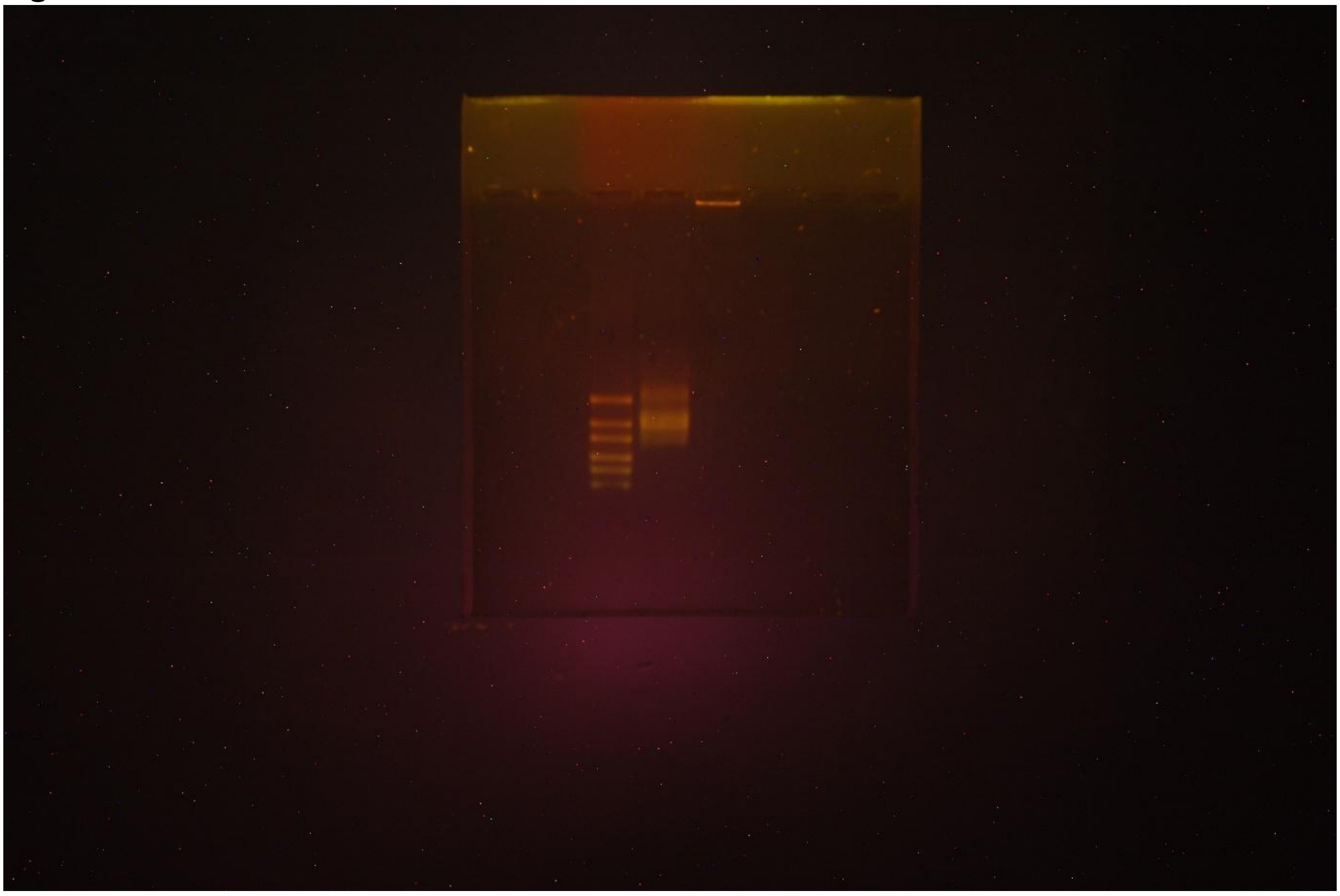

Figure S2b:

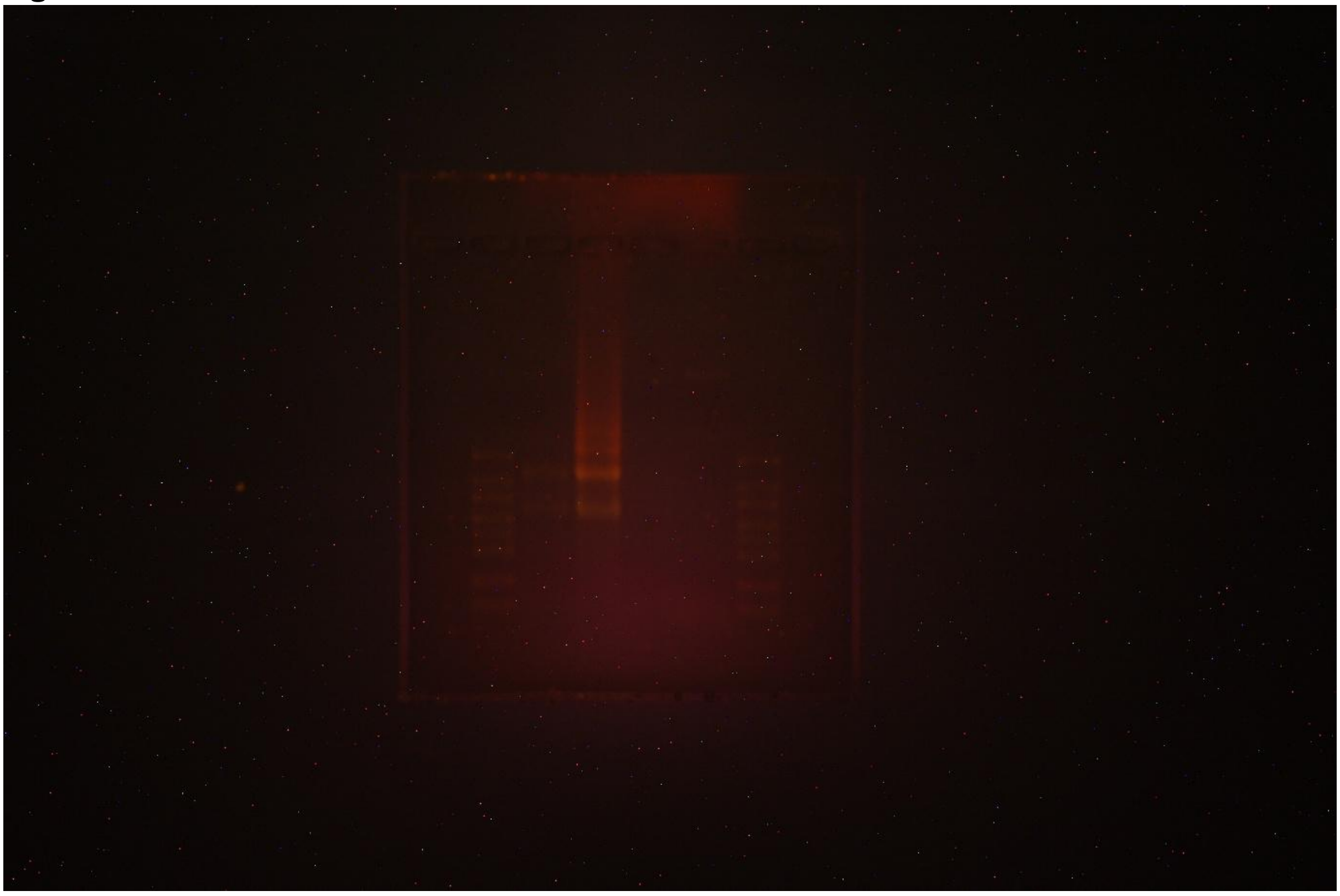

Supplement: Supplementary file 1 [file 41598_2026_55220_MOESM1_ESM.pdf]
